# MTAP-related increased erythroblast proliferation as a mechanism of polycythaemia vera

**DOI:** 10.1038/s41598-021-01877-0

**Published:** 2021-11-18

**Authors:** Chartsiam Tipgomut, Archrob Khuhapinant, Marieangela C. Wilson, Saiphon Poldee, Kate J. Heesom, Chanatip Metheetrairut, Orapan Sripichai, Chalermchai Mitrpant, Jan Frayne, Kongtana Trakarnsanga

**Affiliations:** 1 Department of Biochemistry, Faculty of Medicine Siriraj Hospital, Mahidol University, Bangkok, 10700 Thailand; 2 Division of Haematology, Department of Medicine, Faculty of Medicine Siriraj Hospital, Mahidol University, Bangkok, 10700 Thailand; 3 Proteomics Facility, University of Bristol, Bristol, BS8 1TD UK; 4 National Institute of Health, Department of Medical Sciences, Ministry of Public Health, Nonthaburi, 11000 Thailand; 5 School of Biochemistry, Faculty of Life Sciences, University of Bristol, Bristol, BS81TD UK

**Keywords:** Medical research, Molecular medicine

## Abstract

Polycythaemia vera (PV) is a haematological disorder caused by an overproduction of erythroid cells. To date, the molecular mechanisms involved in the disease pathogenesis are still ambiguous. This study aims to identify aberrantly expressed proteins in erythroblasts of PV patients by utilizing mass spectrometry-based proteomic analysis. Haematopoietic stem cells (HSCs) were isolated from newly-diagnosed PV patients, PV patients who have received cytoreductive therapy, and healthy subjects. In vitro erythroblast expansion confirmed that the isolated HSCs recapitulated the disease phenotype as the number of erythroblasts from newly-diagnosed PV patients was significantly higher than those from the other groups. Proteomic comparison revealed 17 proteins that were differentially expressed in the erythroblasts from the newly-diagnosed PV patients compared to those from healthy subjects, but which were restored to normal levels in the patients who had received cytoreductive therapy. One of these proteins was *S*-methyl-5′-thioadenosine phosphorylase (MTAP), which had reduced expression in PV patients’ erythroblasts. Furthermore, *MTAP* knockdown in normal erythroblasts was shown to enhance their proliferative capacity. Together, this study identifies differentially expressed proteins in erythroblasts of healthy subjects and those of PV patients, indicating that an alteration of protein expression in erythroblasts may be crucial to the pathology of PV.

## Introduction

Polycythaemia vera (PV) is a chronic myeloproliferative neoplasm (MPN) characterised by an overproduction of red blood cells due to increased proliferative capacity of erythroid progenitor cells^1^. The most common symptoms of PV include headache, blurred vision, fatigue, weight loss, pruritus and night sweats, and are mainly due to the hyperviscosity of circulating blood^2^. Advanced stage PV may also contribute to a wide spectrum of thrombotic manifestations and haemorrhages^3^. There are no curative treatments available for PV patients, and the supportive treatments currently available only act to minimise blood viscosity and symptoms by phlebotomy, low-dose aspirin administration and cytoreductive therapy^4^.

*JAK2*^*V617F*^ was identified as a primary mutation in up to 95 percent of PV patients^5–7^. Since then, many studies have examined how *JAK2*^*V617F*^ contributes to the pathological outcomes of PV. Analyses of PV patients with *JAK2*^*V617F*^ suggest that this mutation increases erythroid cell production by increased expansion of erythroid progenitors and erythroblasts^8^. Likewise, mice with conditional expression of *JAK2*^*V617F*^ in haematopoietic cells were also demonstrated to amplify erythroid progenitors through increased cell proliferation^9^. JAK2 mutations promote the constitutive activation of the JAK-STAT pathway, which imitates the physiological responses to cytokine binding, resulting in the expression of genes essential for cell proliferation and survival independent from cytokine availability^10^.

Gene expression profiling in PV received much attention after discovery of JAK2 mutations. A gene expression profiling experiment utilizing cDNA microarray identified altered expression of 644 candidate genes, in particular up-regulation of the transcription factor NF-E2 (nuclear factor, erythroid 2) in granulocytes from PV patients compared to those from healthy individuals^11^. A proteomic analysis of granulocytes from PV patients using two-dimensional gel electrophoresis showed differential expression of 65 proteins in granulocytes from PV patients^12^. Another proteomic study adopted mass spectrometry for systematic evaluation of *JAK2*^*V617F*^ mutation effects in the murine Ba/F3 cell line, identifying over 5000 proteins that were associated with the *JAK2*^*V617F*^ mutation. Notably, pathway analysis of identified proteins demonstrated that many of the changes in protein abundance were regulated by disruption of p53 and MYC signalling pathways^13^. Although a number of proteins have been shown to have abnormal expression patterns in PV patients, the molecular mechanisms that contribute to the pathological characteristics of PV are still unclear.

In the past few years, in vitro erythropoietic culture systems have been progressively improved, providing useful models for studying mechanisms of many red blood cell diseases in a more relevant cell type. In addition, protein expression analysis by mass spectrometry has proved more effective than electrophoresis since this approach provides a way to quantify a large number of proteins without specificity and sensitivity limits. This study therefore aims to compare the proteome of cultured erythroblasts from PV patients and those of healthy individuals using mass spectrometry to elucidate disease pathogenesis and identify proteins that can be adopted for future therapeutic development.

## Results

### Increased proliferative capacity of cultured erythroblasts from PV patients

The proliferative capacity of cultured erythroblasts from newly-diagnosed PV patients (PVN), cytoreductive agent (hydroxyurea)-treated PV patients whose haematocrit had recovered to below the WHO 2016 criteria for PV diagnosis (≤ 49% in men or ≤ 48% in women) (PVT), and healthy subjects (CT) was first identified (n = 3 for each group). All volunteers diagnosed with PV (WHO 2016 criteria for PV diagnosis) tested positive for the *JAK2*^*V617F*^ mutation. More details regarding average age, gender ratio, haematocrit and therapy are summarised in Table 1. The average age of volunteers in the PVT group was higher than those in CT (*p*-value = 0.010) and PVN groups (*p*-value = 0.225) because PV patients should be 60 years old or above to receive cytoreductive agent therapy, according to the WHO guidelines on PV management^14^.

**Table 1:** Detailed information of volunteers included in each group (n = 3 for each group).

Groups	CT	PVT	PVN
Age (year)*	45.33, 38–51	70.33, 63–76	54.00, 35–69
Gender (male–female)	3–0	2–1	3–0
Haemoglobin (g/dl)*	14.7, 14.0–15.5	14.4, 13.6–15.0	18.1, 15.7–21.7
Haematocrit (%)*	43.7, 41.7–45.0	45.2, 43.4—48.3	56.7, 52.1–64.3
Cytoreductive treatment	No	Hydroxyurea	No
Duration of treatment	–	1–3 years	–
Thrombosis history during the treatment	–	No	–

*Mean, range of three individuals.

**Figure 1:**
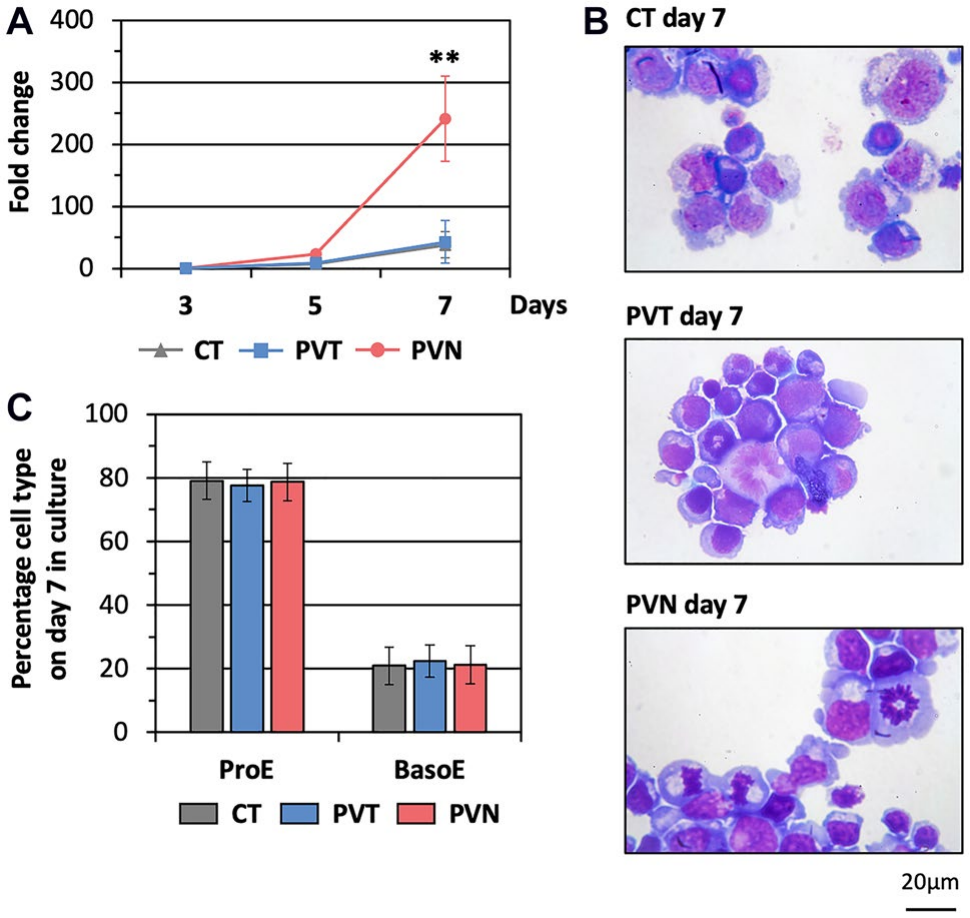
Erythroblasts from PV patients exhibit increased proliferative capacity. Erythroblasts were differentiated from peripheral CD34^+^ cells of newly-diagnosed PV patients (PVN), PV patients who received cytoreductive therapy until their haematocrit was in the normal range (PVT), and healthy subjects as a control group (CT). (**A**) Fold-expansion from day 3 to 7 in culture, mean ± SD of three independent cultures. **p-value < 0.01, ANOVA. (**B**) Morphology of day 7 cultured cells stained with Leishman’s reagent, representative images of three independent cultures, magnification 100 ×. (**C**) Proportion of cells at different erythroid developmental stages on day 7 in culture, mean ± SD of three independent cultures. *ProE* proerythroblast, *BasoE* basophilic erythroblast.

Haematopoietic stem cells (HSCs) isolated from the blood of each volunteer were cultured separately for seven days in a liquid culture system^15^. The number of cells in all PVN cultures started to rise at a greater rate than in both PVT and CT cultures on day 5 and were significantly higher than in the other two groups on day 7 (*p*-value = 0.0081 and 0.0055 compared with CT and PVT groups, respectively; n = 3 for each group). The cell numbers in the PVT and CT cultures were similar during this period in culture (Fig. 1A).

Morphological characterization on day 7 confirmed that the cultured erythroblasts from CT, PVT, and PVN were at a similar developmental stage, the majority (nearly 80%) being proerythroblasts and the remainder basophilic erythroblasts (Fig. 1B,C).

### Proteomics analysis reveals protein expression differences in cultured erythroblasts from PV patients

As the morphology of cultured erythroblasts on day 7 from CT, PVT, and PVN was similar, Tandem Mass Tag (TMT) comparative proteomics was performed at that stage. Samples of day 7 erythroblasts (n = 3) from each group were pooled to reduce individual variations. After filtering all Mass Spectrometry (MS) data to assure a False Discovery Rate (FDR) of 5% and a Peptide-Spectrum Match (PSM) value of more than two, a total of 8330 unique proteins were identified, and the abundance of 8008 unique proteins was compared across the three groups.

Consistent with the morphological characterisation of erythroblasts on day 7, the quantitative MS data showed indistinguishable levels of stage-specific markers for erythroid differentiation, including GYPA, Band 3, CD71, CD36 and alpha 4 integrin, across the three groups (Table 2). In addition, the levels of alpha globin, beta globin and gamma globin were also comparable among all groups which demonstrated validity of the MS dataset (Table 2).

**Table 2:** Relative abundance (log_2_ of fold change) of erythroid proteins in each comparison.

	PVN vs. CT	PVN vs. PVT	PVT vs. CT
**Log**_**2**_** relative abundance**			
CD235a (GYPA)	1.00	0.10	0.91
CD233 (Band 3)	0.65	0.10	0.54
CD71 (TFRC)	0.26	0.15	0.11
CD36	0.01	− 0.22	0.23
CD49d (integrin-α4)	− 0.06	0.00	− 0.06
β-Globin	0.28	− 0.30	0.58
α-Globin	0.36	0.80	− 0.43
γ-Globin	0.16	− 0.76	0.92

#### Pairwise comparison

**Figure 2:**
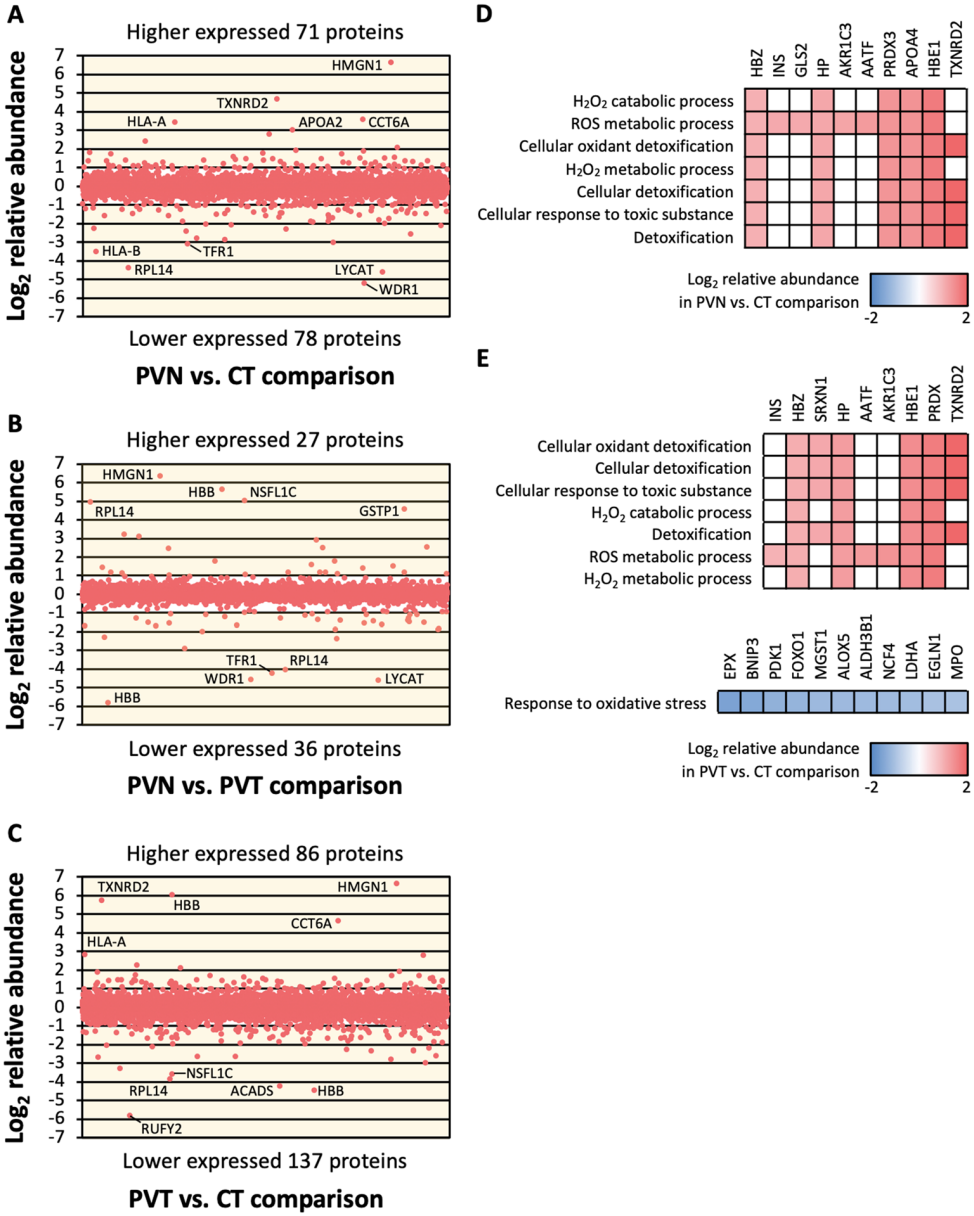
Proteomic analysis of erythroblasts from PV patients reveals protein expression differences. TMT labelling and nano-LC MS/MS were performed on the pooled protein samples prepared from erythroblasts on day 7 in culture. Expression levels of identified proteins were compared across CT, PVT, and PVN. Proteins differentially expressed at two or more-fold in each pairwise comparison were further entered in functional enrichment analysis. (**A**–**C**) Expression patterns of each protein in each pairwise comparison, yellow areas represent protein expression differences at two or more-fold, and names of the top 5 more and less abundant proteins in each comparison were labelled next to their relative abundance. The pairwise comparison shown are (**A**) PVN vs. CT comparison, (**B**) PVN vs. PVT comparison, and (**C**) PVT vs. CT comparison. (**D**) Various processes in cellular oxidant detoxification overrepresented from proteins with increased expression in PVN compared to CT, colour filling indicates proteins apportioned to biological process shown on the left. (**E**) Various processes in cellular oxidant detoxification overrepresented from proteins with increased or decreased expression in PVT compared to CT, colour filling indicates proteins apportioned to biological process shown on the left.

The proteomes of erythroblasts from PVN, PVT, and CT were compared to each other in a pairwise manner in order to identify proteins which may be involved in the molecular mechanism of PV either inclusive of or independent of the increased erythroblast expansion ability observed only for PVN but not for PVT (Fig. 1A). There were 149 proteins differentially expressed at least two-fold in the PVN vs. CT comparison (Fig. 2A, and Table S1 and S2). Additionally, 63 proteins were differentially expressed in the PVN vs. PVT comparison (Fig. 2B, and Table S1 and S2) and 223 proteins were differentially expressed in the PVT vs. CT comparison (Fig. 2C, and Table S1 and S2). Interestingly, among those 223 differentially expressed proteins between PVT vs. CT, 101 proteins were also differentially expressed in the same direction in the PVN vs. CT comparison, suggesting PV erythroid cells are not fully recovered following treatment.

Functional enrichment analysis was performed by g:Profiler^16^. In the PVN vs. CT comparison. Forty-three proteins with increased expression in PVN were included in the classifications based on their molecular function, 10 of which were associated with various processes in cellular oxidant detoxification (Fig. 2D). This finding demonstrates an increase in antioxidant activity in erythroblasts from PVN. Furthermore, functional enrichment analysis was performed on the proteins with increased or decreased expression in PVT compared to CT. Fifty-four and eighty-four proteins with increased and decreased levels in PVT were included in the classifications based on their molecular function, respectively. Similar to the PVN vs. CT comparison, 9 proteins with roles in various cellular oxidant detoxification processes were increased in level in PVT cells (Fig. 2E), 7 of which were common to those expressed at a higher level in PVN compared to CT. However, eleven proteins at a decreased level in PVT were also associated with the oxidative stress response (Fig. 2E).

Functional enrichment analyses of proteins at decreased levels in PVN when compared to CT or with increased or decreased levels in PVN compared to PVT were also performed by g:Profiler but there were no results with FDR of 5% enriched from the analysis.

#### Comparison of proteome of erythroblasts with normal vs. abnormal expansion capacity

**Figure 3:**
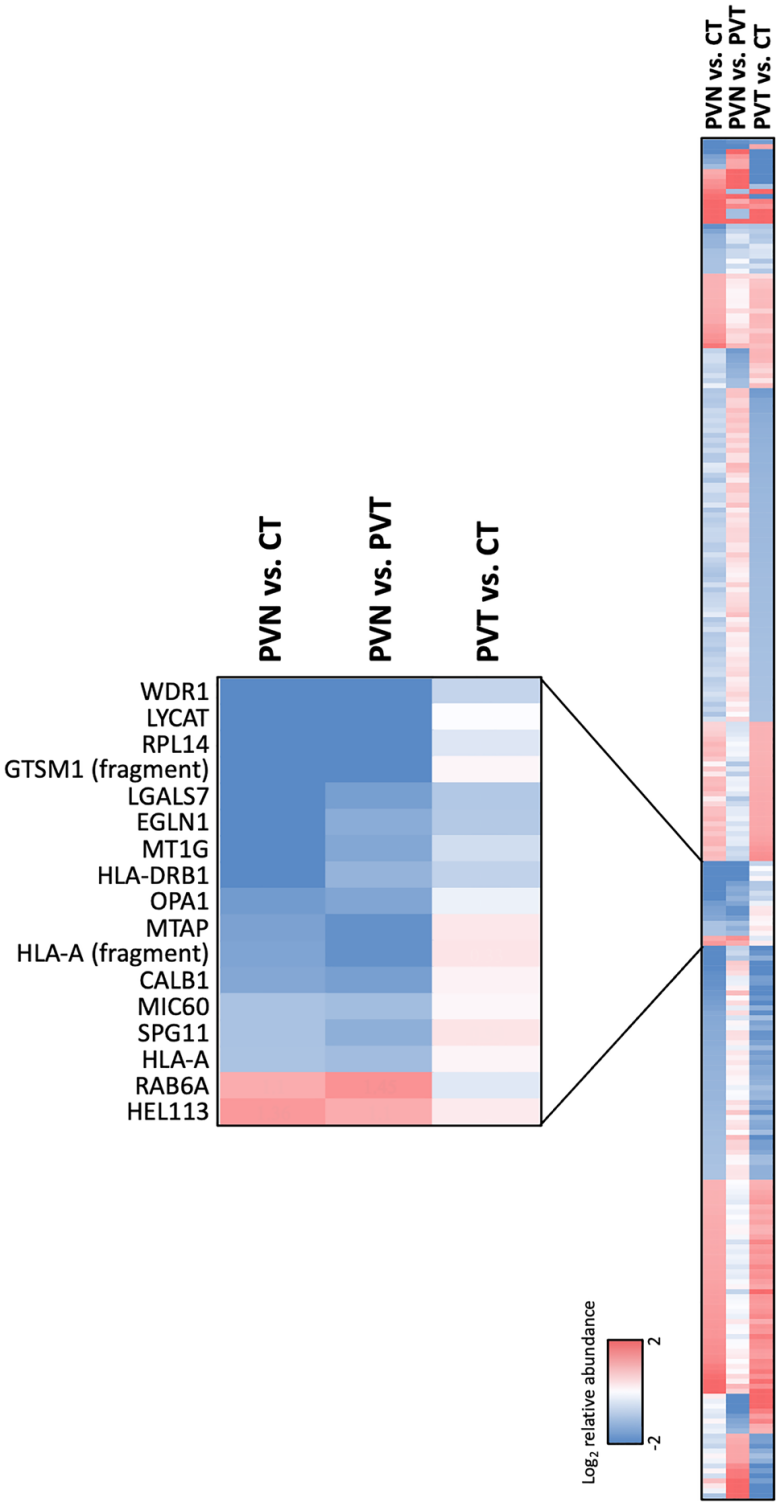
Expression heatmap of the differentially expressed proteins. TMT labelling and nano-LC MS/MS were performed on the pooled protein samples prepared from erythroblasts on day 7 in culture. Expression levels of identified proteins were compared across CT, PVT, and PVN. Proteins higher or lower expressed two or more-fold in PVN vs. CT, PVN vs. PVT, or PVT vs. CT comparisons were plotted on the heatmap, magnified area demonstrated 17 differentially expressed proteins in both PVN vs. CT and PVN vs. PVT comparisons but not in the PVT vs. CT comparison.

The in vitro erythroblasts from PVN patients had a greater expansion rate compared to erythroblasts from both PVT patients and controls (Fig. 1A). Therefore, all proteomic comparisons were combined to identify proteins responsible for the increased expansion of the PVN erythroblasts. Although there were 297 proteins at higher or lower levels by two or more-fold in at least one comparison (Table S1), there were only 17 proteins whose levels differed in PVN compared to both CT and PVT, but were approximately the same in PVT and CT (Fig. 3). Several of these, including *S*-methyl-5′-thioadenosine phosphorylase (MTAP)^17^, EGL-nine 1 (EGLN1)^18^, and metallothionein 1G (MT1G)^19^ have been demonstrated to have a role in either cell proliferation or cancer development.

To further validate the quantitative MS data, western blot analysis was performed for selected proteins using the protein samples from day 7 erythroblasts. The abundance of β-globin was used to normalise the protein expression levels. Similar to abundance profiles in the MS data, the levels of Band3 and NF-E2 were similar in CT, PVT and PVN samples, whereas the level of MTAP was lower in PVN compared to CT and PVT samples (Figure S1 and Table 3).

**Table 3:** Relative abundances (log_2_ of fold change) of Band 3, MTAP and NF-E2 from quantitated western blot band intensities (normalised with β-globin) and MS data.

	PVN vs. CT		PVN vs. PVT		PVT vs. CT	
	WB	MS	WB	MS	WB	MS
Band3	0.98	1.57	1.10	1.07	0.89	1.46
MTAP	0.41	0.34	0.37	0.27	1.11	0.80
NF-E2	1.00	0.98	0.84	0.96	1.21	1.03

### MTAP knockdown enhances proliferative capacity in normal erythroblasts

**Figure 4:**
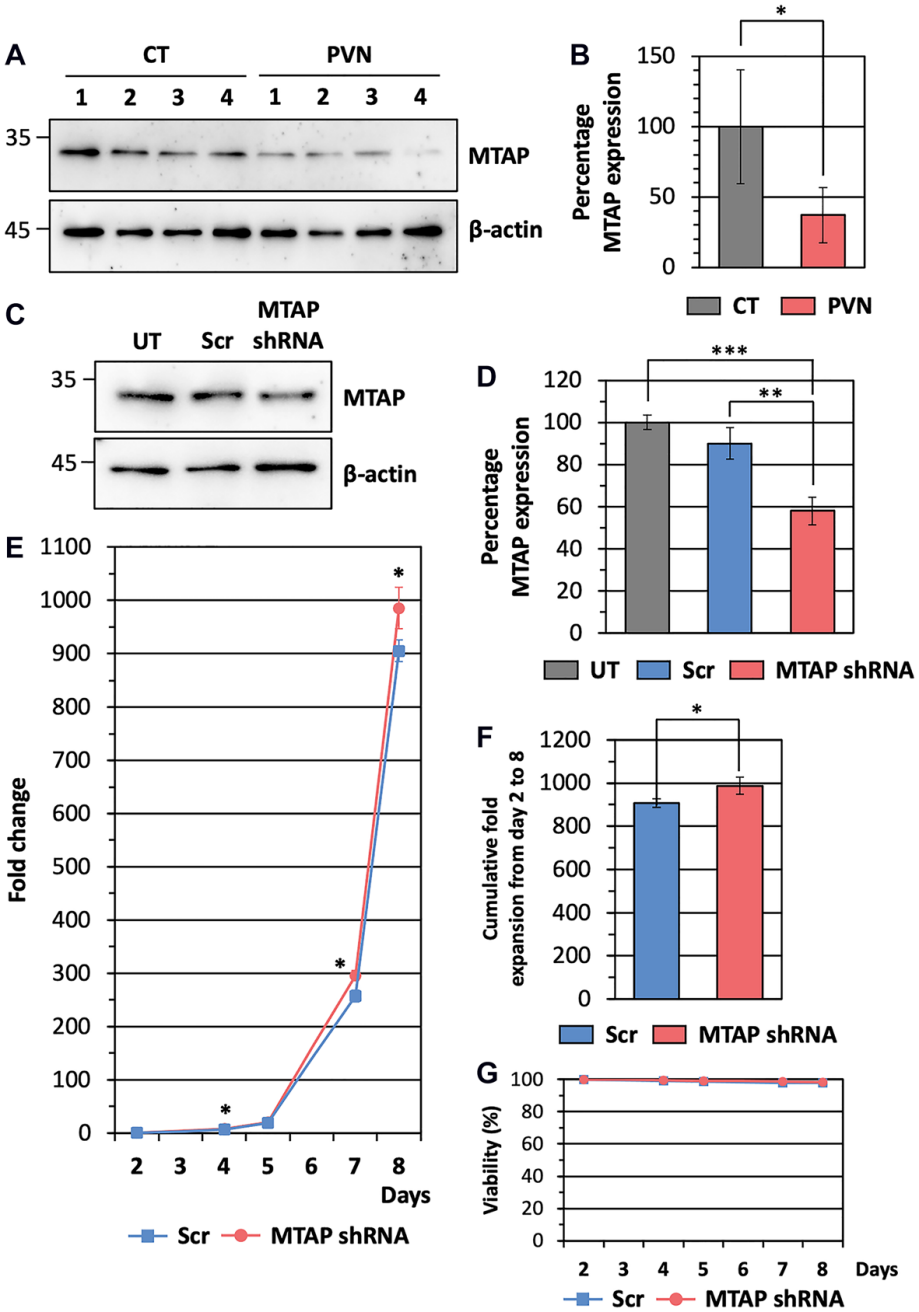
*MTAP* knockdown enhances proliferative capacity in normal erythroblasts. (**A**) Representative visualised bands of MTAP and a loading control β-actin from western blot of day 7 erythroid cells from newly-diagnosed PV patients (PVN) and healthy subjects (CT). (**B**) Density of MTAP bands on Western blots (in **A**) were quantified using ImageJ normalized to β-actin, mean ± SD of four independent cultures. (**C**–**G**) Erythroblasts differentiated from adult peripheral CD34^+^ cells were transduced with MTAP shRNA or control scrambled shRNA (Scr) on day 2 in culture. Untransduced cells (UT) served as a control. (**C**) Representative visualised bands of MTAP and a loading control β-actin from western blot at 72 h post-transduction. (**D**) Density of MTAP bands on Western blots (in **C**) were quantified using ImageJ normalized to β-actin, mean ± SD of three independent cultures. (**E**) Fold-expansion from day 2 to 8 in culture, mean ± SD of three independent cultures. (**F**) Cumulative fold expansion from day 2 to 8 in culture, mean ± SD of three independent cultures. (**G**) Viability from day 2 to 8 in culture, mean ± SD of three independent cultures. *p-value < 0.05, **p-value < 0.01, two-tailed *t* test.

MTAP is a crucial mammalian enzyme that catalyses the cleavage of *S*-methyl-5′-thioadenosine (MTA), a by-product of polyamine biosynthesis^20^. Loss of MTAP and the accumulation of MTA have been shown to support a wide variety of malignancies, as reported by many clinical investigations of patients with different types of solid tumour and acute type of adult T-cell leukaemia^17^. Furthermore, *JAK2*^*V617F*^, a primary mutation in PV, has been shown to constitutively induce the expression of MYC and its downstream target genes, including ornithine decarboxylase (ODC) which is also an enzyme in the polyamine metabolism pathway^21^. Since our MS data also showed a 2.99-fold decrease in MTAP expression in erythroblasts from PVN compared to those from CT, MTAP may be a factor that regulates the increased expansion of erythroblasts in PV patients. Similar to the MS data, MTAP levels in erythroblasts from PV patients, analysed by western blot, were significantly lower than those of healthy individuals (*p*-value = 0.0311; n = 4) (Fig. 4A,B). In order to explore the relationship between MTAP expression and erythroblast proliferation, MTAP expression was reduced in normal erythroblasts by shRNA. Cultured erythroblasts from healthy subjects were transduced with MTAP shRNA or control scrambled shRNA (Scr) on day 2 in culture. At 72 h post-transduction, erythroblasts transduced with MTAP shRNA have significantly decreased MTAP expression compared to those transduced with Scr (*p*-value = 0.0006; n = 3) and the untransduced control (*p*-value = 0.0050; n = 3) (Fig. 4C,D).

The number of MTAP shRNA transduced erythroblasts were significantly higher than those of Scr transduced erythroblasts on days 4, 7, and 8 (Fig. 4E), yielding higher cumulative fold expansion from the MTAP shRNA transduced erythroblast cultures (*p*-value = 0.0349; n = 3) (Fig. 4F). *MTAP* knockdown did not affect the viability of erythroblasts (Fig. 4G).

### MTAP knockdown does not influence erythroid differentiation

**Figure 5:**
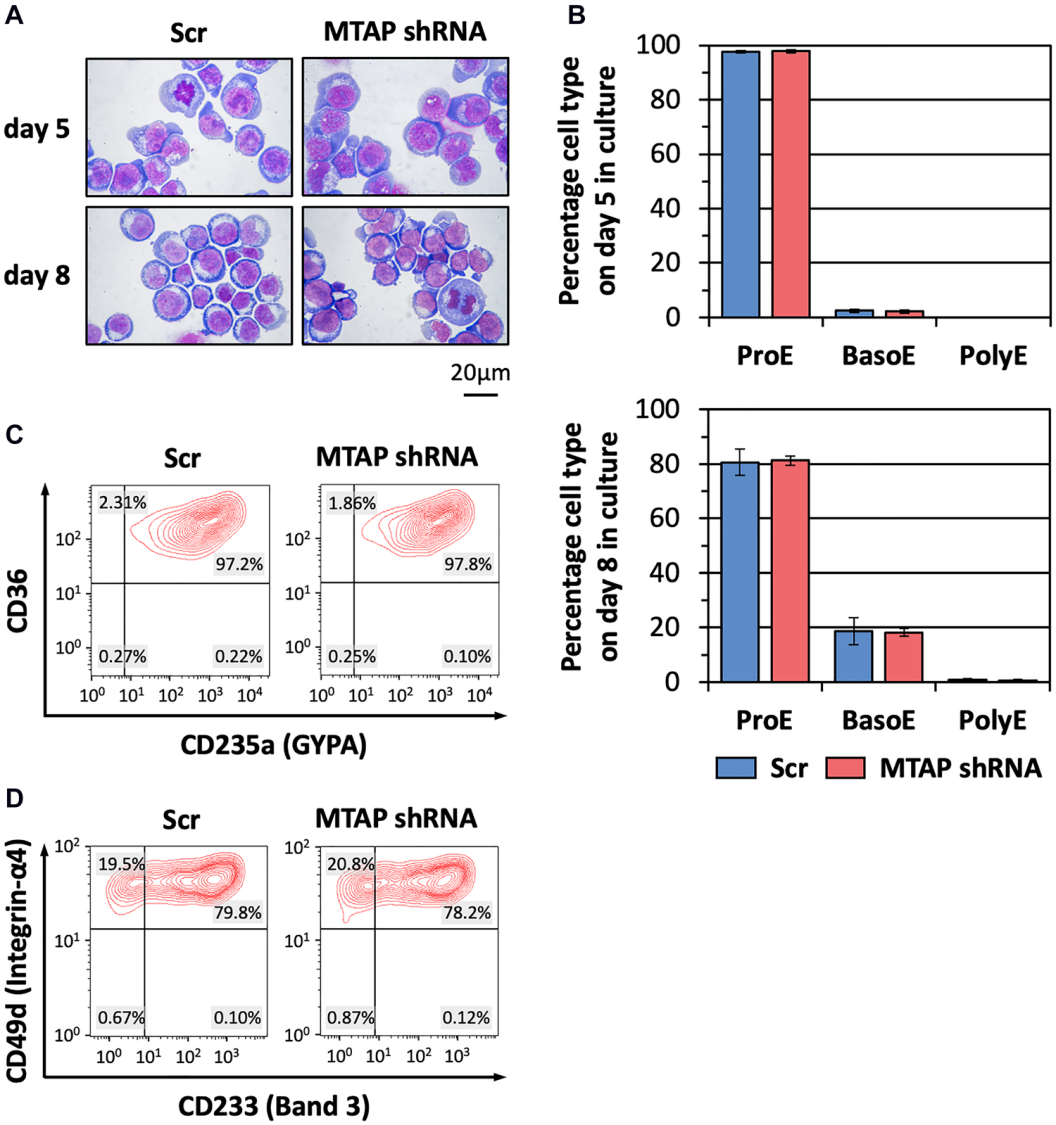
Erythroid differentiation is unaffected by *MTAP* knockdown. Differentiation of erythroblasts transduced with MTAP shRNA or control scrambled shRNA (Scr) was examined at specific time points. (**A**) Morphology of cultured cells stained with Leishman’s reagent on days 5 and 8, representative images of three independent cultures, magnification 100 ×. (**B**) Composition of cell types at different erythroid developmental stages on days 5 and 8 in culture, mean ± SD of three independent cultures, *ProE* proerythroblast, *BasoE* basophilic erythroblast, *PolyE* polychromatic erythroblast. (**C**) Expression of membrane markers CD235a (GYPA) vs. CD36 on day 8 analysed by flow cytometry. (**D**) Expression of membrane markers CD233 (Band 3) vs. CD49d (Integrin-α4) on day 8 analysed by flow cytometry.

Morphological characterization demonstrated that erythroblasts transduced with MTAP shRNA had a similar differentiation profile to those transduced with Scr shRNA. All cells in both groups were proerythroblasts on day 5, which later differentiated down the erythroid lineage, yielding approximately 19% basophilic erythroblasts on day 8 (Fig. 5A,B). In addition, flow cytometric analysis on day 8 erythroblasts shows similar patterns, which were high CD235a (glycophorin A; GYPA) with high CD36 and high CD49d (Integrin-α4) with varying amount of CD233 (Band 3) (Fig. 5C,D). These results confirm that the rate of erythroid differentiation of erythroblasts transduced with MTAP shRNA and those transduced with Scr shRNA were similar.

## Discussion

In vitro culture of erythroid cells differentiated from HSC of newly-diagnosed PV patients showed a significantly higher rate of expansion of early erythroblasts compared to those of treated PV patients and controls, recapitulating the major disease phenotype of PV, which is an overproduction of RBCs^2^. Although cytoreductive agents used for PV treatment were omitted in the culture system for erythroblasts from PV patients who had received cytoreductive treatment, the cell numbers during days 3 to 7 between PVT and CT groups were similar. This finding concurs well with a previous study that has demonstrated an influenced cell cycle status of cord blood CD34^+^ HSCs after being pre-exposed to hydroxyurea^22^. Therefore, it could be conceivably assumed that prior cytoreductive treatment may affect HSCs of PV patients, resulting in decreased cell numbers observed in PVT.

Previously, *JAK2*^*V617F*^ was found to stimulate ROS-dependent oxidative stress in an in vitro mouse model and in cultured patient cells^23,24^. However, erythroblasts from PV patients with *JAK2*^*V617F*^ are not affected by oxidative cell death. An explanation proposed is that the patients’ erythroblasts may up-regulate antioxidant defence mechanisms to avoid extensive damage from oxidative stress^25^. This concept is consistent with our functional enrichment analysis results, which identified increased levels of proteins associated with various processes in cellular oxidant detoxification in erythroblasts from PV patients compared to healthy subjects (Fig. 2D). Seven out of these ten increased proteins were also increased in erythroblasts from PV patients who received cytoreduction therapy (Fig. 2E), which indicates that the increase in cellular oxidant detoxification activity is a characteristic of erythroblasts from PV patients. The finding of eleven proteins at decreased levels in erythroblasts from patients who received cytoreduction therapy might be due to an effect of the therapy. Further studies, including ROS quantification in these cells, need to be performed to verify this finding.

In contrast to previous gene expression profiling that found both mRNA and protein levels of transcription factor NF-E2 to be overexpressed in granulocytes from PV patients^11^, the quantitative data from MS and western blot in the present study demonstrated no change in NF-E2 levels in cultured erythroblasts from the three groups (Fig. S1 and Table 3). This may be due to the differential regulation of NF-E2 in erythroid cells compared to granulocytes and thus different downstream effects of the mutation.

Integrative analysis of proteomic comparisons identified 17 proteins, the levels of which differed in PVN compared to CT and PVT, but was approximately the same in PVT and CT. This finding suggests that these 17 proteins might be abnormally expressed in erythroblasts from untreated PV patients, and that their expression levels may return to normal after cytoreductive therapy. Notably, aberrant expression of three proteins among these has been highlighted as being involved in cell proliferation and tumour growth, in particular MTAP down-regulation^17,26,27^. Interestingly, the polyamine metabolism pathway, in which MTAP functions, has also been shown to be regulated by JAK2 as the constitutively active *JAK2*^*V617F*^ mutation leads to constitutive expression of ODC, a rate-limiting enzyme in this pathway^21^. Polyamine molecules were reported to be higher in plasma of PV patients than in that of healthy controls^28^, though there have been no reports in erythroid cells so far. These results indicate a possible involvement of the polyamine metabolism pathway in *JAK2*^*V617F*^-induced PV pathogenesis, suggesting that MTAP, as part of the pathway, may be a candidate of interest. The in vitro functional analysis of MTAP down-regulation in the present study, which showed increased erythroid cell expansion, supports a role for MTAP in the PV disease phenotype. However, the increase in cell numbers in *MTAP* knockdown erythroblasts from days 3 to 7 was 3.26-fold lower than observed in the cultured erythroblasts from PV patients (Fig. 1A). This difference could be explained in part by other proteins that may be required to act co-operatively with the MTAP down-regulation to enhance proliferative capacity and establish overproduction of erythroid cells. Another possible reason is that the reduction in MTAP expression in *MTAP* knockdown erythroblasts (1.69-fold decrease compared with the scrambled shRNA control) is lower than that in erythroblasts from PV patients (2.99-fold decrease compared with erythroblasts from healthy subjects).

Socoro-Yuste et al. identified 67 proteins in red blood cells from PV patients that had altered levels compared to those of healthy individuals^29^. However, these proteins were not at an altered level in the proteomic analysis in the present study. One possible explanation for these contradictory results is that the cell used were at different erythroid developmental stages, and the expression of many proteins in early erythroid cells is typically changed during maturation^30^.

Previous gene expression analysis by oligonucleotide microarray has revealed that men with PV have more differentially expressed genes than women, suggesting that gender may be a confounding factor in expression profiling^31^. Also, PV pathogenesis, phenotype, and prognosis are all considered to be gender-biased^32^. Unfortunately, the gender of our patient cohort was predominantly men, which reflects the higher prevalence of PV in males but may confound protein expression studies. While our functional experiment on MTAP has highlighted its role in promoting erythroid proliferation, additional data collection on PV patients stratified by gender is, therefore, required for translational research. In addition, JAK2(V617F) allele burden has been shown to be associated with severity of the disease^33,34^. It would be interesting to know whether the protein deregulation identified in this study is related to JAK2(V617F) allele burden. Further proteomic study in patients with known JAK2(V617F) allele burden should be performed.

In conclusion, this study examines the proteome of erythroid cells associated with the PV pathogenesis by utilising an MS-based proteomic approach for qualitative and quantitative analysis, providing further insight into proteins involved in this disease. Identification of 149 differentially expressed proteins in erythroblasts from PV patients and those of healthy subjects, suggest that changes in such protein expression could collectively contribute to the PV pathology. Last but not least, the implications of this study regarding aberrant protein expression could potentially define rational targets for novel treatments.

## Methods

### Cell isolation and culture

Whole blood was obtained from healthy donors (without G-CSF stimulation) and PV patients with written informed consent for research use following the Declaration of Helsinki and approved by Siriraj Institutional Review Board (SIRB) (Certificate of Approval (COA) number: SI190/2016). Peripheral CD34^+^ cells were isolated from whole blood and cultured in the three-stage erythroid culture system described previously by Griffiths et al*.*^15^. Briefly, the isolated CD34^+^ cells were maintained in Iscove’s medium (Gibco) containing 3% (v/v) human AB serum (Sigma), 2% (v/v) FBS (Sigma), 10 µg/ml insulin (Sigma), 3 U/ml heparin (Sigma), 3 U/ml Epoetin-β (Roche), 10 ng/ml SCF (R&D Systems), 1 ng/ml IL-3 (R&D Systems), 200 µg/ml holo-transferrin (R&D Systems), and 1 U/ml pen/strep (Sigma). The cultured cells were counted every other day, and fresh medium was added to obtain a final concentration of 1 to 2 × 10^5^ cells/ml. The cultured cells were maintained in tissue culture flasks at 37 °C and 5% CO_2_ throughout the culture period.

### Total protein lysate preparation

The cultured cells from each subject were harvested and frozen as pellet on day 7 of culture. The frozen cell pellet was thawed on ice and resuspended in solubilization buffer (20 mM Tris–HCl pH 7.5, 150 mM NaCl, 10% (v/v) glycerol, 1% (v/v) Triton^TM^ X-100, 0.1% (w/v) SDS) containing Complete protease inhibitor cocktail (Roche). After centrifugation, protein concentrations were measured using the protein assay dye reagent (BioRad).

### Comparative proteomics

50 µg of protein from each individual from the same group were pooled together to reduce variation among individuals. TMT-based MS/MS analysis was performed as described previously^35^. Briefly, aliquots of 50 µg protein from each group were digested with trypsin (1.25 µg trypsin per 50 µg protein) at 37 °C overnight. The digested samples were then labelled with TMT10plex™ isobaric mass tagging reagents, and the labelled samples were pooled. The pooled sample was desalted and fractionated by high pH reversed-phase chromatography using an Ultimate 3000 liquid chromatography system (Thermo Fisher Scientific). The resulting fractions were evaporated to dryness and resuspended in 1% formic acid prior to analysis by nano-LC MSMS using an Orbitrap Fusion Tribrid mass spectrometer (Thermo Scientific). All spectra were acquired using an Orbitrap Fusion Tribrid mass spectrometer controlled by Xcalibur 2.0 software (Thermo Scientific) and operated in data-dependent acquisition mode using an SPS-MS3 workflow.

### Proteomic data analysis

The raw data files were processed and quantified using Proteome Discoverer 2.1 and searched against the UniProt Human database using the SEQUEST HT algorithm. All data were filtered to satisfy the false discovery rate (FDR) of 5%, and identified proteins with two or more PSMs were included in further analysis. The functional enrichment analysis of differentially expressed proteins was performed using g:Profiler (version e103_eg50_p15_68c0e33) with g:SCS multiple testing correction method applying significance threshold of 0.05^16^.

### SDS-PAGE and western blot

Proteins were resolved by SDS-PAGE and transferred to PVDF (Millipore). Membranes were blocked with 10% milk powder followed by incubation with primary antibodies against β-globin (Santa Cruz), Band 3 (IBGRL), MTAP, NF-E2 (both from Abcam) or β-actin (Sigma). Secondary antibodies were goat α-rabbit IgG-HRP and rat α-mouse IgG1-HRP (Abcam). Membranes were incubated with Immobilon Crescendo western HRP substrate (Merck), and bands were visualised by ImageQuant LAS 4000 (GE Life Sciences). For protein quantitation, the intensities of visualised bands were obtained from ImageJ 1.52.

### Flow cytometry

Cultured cells at selected time points were harvested and washed once in PBSAG (PBS containing 1 mg ml^−1^ BSA and 2 mg ml^−1^ glucose). The cell pellet was then resuspended and incubated with 1:1 dilution of respective primary antibodies against CD233 or CD235a (IBGRL) for 60 min at 4 °C. The cells were washed once in PBS-AG and incubated with 1:500 dilution of rat α-mouse IgG1-APC (Biolegend) for 30 min at 4 °C, followed by washing as above. For dual staining, cells were co-stained with 1:10 dilution of desired FITC-conjugated antibodies against CD36 and CD49d (Biolegend) for 30 min at 4 °C. Flow cytometric data was acquired by FACSCalibur (Becton Dickinson) and analysed by FlowJo 10.

### Lentiviral transduction

For *MTAP* knockdown, day 2 pro-erythroblasts differentiated from normal peripheral blood CD34^+^ cells were transduced with pLKO.1 shRNA plasmid TRCN0000256255 against *MTAP* or a scrambled control shRNA (both designed by the Broad Institute and purchased from Sigma-Aldrich) in the presence of 8 μg ml^−1^ polybrene.

### Statistical analyses

Statistical analyses were performed using Microsoft Excel 2019 (Microsoft). Data are expressed as mean ± SD. Statistical significance was determined by ANOVA or two-tailed unpaired *t* test. p value of < 0.05 was considered statistically significant.

## Supplementary Information

Supplementary Information.
