# Supplementary material for: MTAP-related increased erythroblast proliferation as a mechanism of polycythaemia vera
Source: Sci Rep. 2021 Nov 18;11:22483. doi: 10.1038/s41598-021-01877-0 (PMC8602418; doi:10.1038/s41598-021-01877-0)
Supplement: Supplementary file 1 — Supplementary Information. [file 41598_2021_1877_MOESM1_ESM.docx]

**MTAP-related Increased Erythroblast Proliferation as a Mechanism of Polycythaemia Vera**

Chartsiam Tipgomut^1^, Archrob Khuhapinant^2^, Marieangela C. Wilson^3^, Saiphon Poldee^1^, Kate J. Heesom^3^, Chanatip Metheetrairut^1^, Orapan Sripichai^4^, Chalermchai Mitrpant^1^, Jan Frayne^5^, Kongtana Trakarnsanga^1*^

^1^Department of Biochemistry, Faculty of Medicine Siriraj Hospital, Mahidol University, Bangkok 10700, Thailand.

^2^Division of Haematology, Department of Medicine, Faculty of Medicine Siriraj Hospital, Mahidol University, Bangkok 10700, Thailand.

^3^Proteomics Facility, University of Bristol, Bristol BS8 1TD, UK.

^4^National Institute of Health, Department of Medical Sciences, Ministry of Public Health, Nonthaburi 11000, Thailand.

^5^School of Biochemistry, Faculty of Life Sciences, University of Bristol, Bristol BS81TD, UK.

*Correspondence: [Kongtana.tra@mahidol.ac.th](mailto:Kongtana.tra@mahidol.ac.th)

**Table S1** Proteins differentially expressed two or more-fold in at least one comparison

| **Protein name** | **Log_2_**  **PVN vs. CT** | **Log_2_**  **PVN vs. PVT** | **Log_2_ P**  **VT vs. CT** |
| --- | --- | --- | --- |
| HLA-B | -3.49 | -1.85 | -1.64 |
| cDNA FLJ75881 | -3.1 | -4.21 | 1.13 |
| RUFY2 | -2.87 | 2.92 | -5.8 |
| DPP4 | -1.58 | 1.4 | -2.99 |
| HLA-A | -1.44 | 1.15 | -2.59 |
| CXCR4 | -1.12 | 1.19 | -2.31 |
| cDNA FLJ76611 | 1.11 | 4.96 | -3.84 |
| HBB (variant fragment) | 1.21 | 5.66 | -4.44 |
| NSFL1C | 1.44 | 5.03 | -3.59 |
| Carboxypeptidase | 1.52 | 2.56 | -1.04 |
| ELOF1 | 1.76 | -1.04 | 2.8 |
| GSTP1 (fragment) | 1.91 | 4.59 | -2.68 |
| APOC4-APOC2 | 2.79 | 1.09 | 1.7 |
| APOA2 | 3.03 | 1.6 | 1.43 |
| CCT6A (fragment) | 3.59 | -1.08 | 4.67 |
| TXNRD2 | 4.68 | -1.06 | 5.75 |
| HMGN1 | 6.64 | 6.35 | 6.64 |
| Sulfotransferase | -1.88 | -0.91 | -0.97 |
| cDNA FLJ77684 | -1.52 | -0.69 | -0.82 |
| LTB4DH | -1.27 | -0.32 | -0.94 |
| PER2 | -1.25 | -0.42 | -0.84 |
| PRRC2A | -1.23 | -0.89 | -0.34 |
| MT1X | -1.05 | -0.6 | -0.45 |
| NLRP2 | -1.03 | -0.61 | -0.42 |
| cDNA FLJ35817 fis | -1.01 | -0.06 | -0.95 |
| HLA-B (fragment) | -1.01 | -0.56 | -0.45 |
| NIPSNAP1 | -1 | -0.09 | -0.91 |
| APOC1 | 1.01 | 0.54 | 0.46 |
| SLC6A9 | 1.01 | 0.26 | 0.75 |
| C4A | 1.01 | 0.21 | 0.8 |
| GYPA | 1.01 | 0.1 | 0.91 |
| cDNA FLJ52464 | 1.02 | 0.11 | 0.91 |
| GNL3 | 1.03 | 0.14 | 0.9 |
| cDNA FLJ25861 fis | 1.03 | 0.11 | 0.92 |
| HSpTB1 (fragment) | 1.08 | 0.47 | 0.61 |
| DDX56 | 1.11 | 0.14 | 0.96 |
| GLS2 | 1.12 | 0.17 | 0.95 |
| cDNA FLJ38861 fis | 1.26 | 0.33 | 0.92 |
| DEFA1 | 1.31 | 0.61 | 0.7 |
| APOA4 | 1.42 | 0.45 | 0.97 |
| CCDC18 | 1.49 | 0.5 | 0.99 |
| SAA2-SAA4 | 1.79 | 0.95 | 0.84 |
| TRAP1 (fragment) | -0.71 | -1.69 | 0.97 |
| GTSF1 | -0.52 | -1.51 | 0.99 |
| ADAM10 | -0.51 | -1.46 | 0.94 |
| cDNA FLJ53399 | -0.68 | -1.38 | 0.7 |
| Q8TA96_HUMAN (fragment) | -0.74 | -1.27 | 0.53 |
| cDNA, FLJ93605 | -0.49 | -1.25 | 0.76 |
| GGACT | -0.75 | -1.08 | 0.34 |
| CDH15 | -0.19 | -1.06 | 0.86 |
| HLA-A | -0.92 | 0.78 | -1.7 |
| SCD (variant fragment) | -0.85 | 0.8 | -1.66 |
| CLC | -0.93 | 0.55 | -1.49 |
| ENO2 | -0.87 | 0.59 | -1.46 |
| RAF3 | -0.6 | 0.85 | -1.44 |
| AKAP12 | -0.68 | 0.7 | -1.38 |
| RAB3D | -0.55 | 0.82 | -1.38 |
| RASSF2 | -0.68 | 0.65 | -1.34 |
| ITGB2 | -0.54 | 0.78 | -1.32 |
| PDK1 | -0.88 | 0.44 | -1.31 |
| RSF1 | -0.65 | 0.67 | -1.31 |
| ZNF395 | -0.85 | 0.44 | -1.29 |
| FOXG1 | -0.57 | 0.71 | -1.28 |
| PGM1 | -0.88 | 0.38 | -1.26 |
| P4HA1 (fragment) | -0.86 | 0.4 | -1.26 |
| FOXO1 | -0.31 | 0.95 | -1.26 |
| SLC25A21 | -0.41 | 0.84 | -1.25 |
| ACSL4 | -0.8 | 0.44 | -1.23 |
| GBE1 (variant fragment) | -0.99 | 0.24 | -1.22 |
| GATA1 | -0.49 | 0.72 | -1.21 |
| SCARB1 | -0.48 | 0.73 | -1.21 |
| LDHD | -0.7 | 0.49 | -1.19 |
| NCF1B | -0.67 | 0.52 | -1.19 |
| TBL1X | -0.38 | 0.81 | -1.19 |
| ANXA6 | -0.97 | 0.21 | -1.18 |
| RAB44 | -0.7 | 0.48 | -1.18 |
| cDNA FLJ94534 | -0.84 | 0.33 | -1.17 |
| PF4 | -0.74 | 0.43 | -1.17 |
| MYO1G | -0.63 | 0.54 | -1.17 |
| ALOX5 | -0.66 | 0.51 | -1.16 |
| Terpene cyclase/mutase family member | -0.65 | 0.52 | -1.16 |
| UBTF | -0.83 | 0.31 | -1.15 |
| ALDH3B1 | -0.8 | 0.35 | -1.15 |
| VWA5A | -0.47 | 0.68 | -1.15 |
| cDNA FLJ16598 fis | -0.69 | 0.45 | -1.14 |
| cDNA FLJ78063 | -0.81 | 0.32 | -1.13 |
| FLNB | -0.92 | 0.21 | -1.12 |
| cDNA FLJ95841 | -0.99 | 0.13 | -1.11 |
| PGK1 | -0.87 | 0.24 | -1.11 |
| GPSM1 | -0.5 | 0.61 | -1.11 |
| EPCAM | -0.92 | 0.19 | -1.1 |
| JAZF1 | -0.69 | 0.41 | -1.1 |
| LPCAT2 | -0.64 | 0.45 | -1.1 |
| LDHA | -0.61 | 0.5 | -1.1 |
| IL16 | -0.49 | 0.61 | -1.1 |
| CPA3 | -0.27 | 0.82 | -1.09 |
| B4GALT1 | -0.92 | 0.16 | -1.08 |
| cDNA FLJ92490 | -0.72 | 0.35 | -1.08 |
| ITGA2B | -0.49 | 0.59 | -1.08 |
| A2M | -0.9 | 0.17 | -1.07 |
| CD37 | -0.82 | 0.25 | -1.07 |
| SLC2A3 | -0.77 | 0.29 | -1.07 |
| cDNA FLJ78298 | -0.94 | 0.11 | -1.05 |
| MPI | -0.81 | 0.24 | -1.05 |
| LHPP | -0.66 | 0.39 | -1.05 |
| SLAF5 | -0.72 | 0.32 | -1.04 |
| APBB1IP | -0.6 | 0.44 | -1.04 |
| P2RX4 | -0.52 | 0.52 | -1.04 |
| CTSS | -0.82 | 0.2 | -1.03 |
| PHGDH | -0.74 | 0.29 | -1.03 |
| NEFH | -0.63 | 0.4 | -1.03 |
| MSMO1 | -0.46 | 0.57 | -1.03 |
| EGLN1 | -0.63 | 0.39 | -1.02 |
| MPO | -0.9 | 0.11 | -1.01 |
| PGK2 | -0.66 | 0.35 | -1.01 |
| HLA-DRB1 | -0.97 | 0.03 | -1 |
| PRG1 | -0.47 | 0.54 | -1 |
| TXLNG | 0.55 | -0.46 | 1.01 |
| NOP16 | 0.58 | -0.43 | 1.01 |
| ALYREF | 0.65 | -0.35 | 1.01 |
| DKFZp686I15212 | 0.81 | -0.2 | 1.01 |
| FTH1 (fragment) | 0.96 | -0.05 | 1.01 |
| HIST1H1C | 0.84 | -0.17 | 1.02 |
| DKFZp686I04196 (fragment) | 0.93 | -0.09 | 1.02 |
| MYH10 | 0.71 | -0.33 | 1.03 |
| SEPT1 | 0.12 | -0.92 | 1.04 |
| PPIP5K1 | 0.75 | -0.32 | 1.06 |
| ATP2B2 | 0.28 | -0.79 | 1.07 |
| GNL2 | 0.91 | -0.17 | 1.08 |
| HIST1H1B | 0.87 | -0.23 | 1.09 |
| DGKB | 0.98 | -0.12 | 1.11 |
| RRP7A | 0.75 | -0.37 | 1.12 |
| PIP | 0.21 | -0.92 | 1.13 |
| SRXN1 | 0.54 | -0.59 | 1.13 |
| cDNA FLJ76883 | 0.83 | -0.3 | 1.13 |
| SOS2 | 0.83 | -0.3 | 1.13 |
| Uncharacterized protein (Q8TCD0_HUMAN) | 0.96 | -0.17 | 1.13 |
| AKR1C2 | 0.67 | -0.48 | 1.15 |
| cDNA FLJ77835 | 0.91 | -0.24 | 1.15 |
| SERBP1 | 0.74 | -0.45 | 1.18 |
| LLPH | 0.96 | -0.27 | 1.24 |
| HIST1H1E | 1 | -0.31 | 1.3 |
| ODC1 | 0.73 | -0.66 | 1.39 |
| MKI67 (fragment) | 0.92 | -0.54 | 1.46 |
| ZNF593 | 0.83 | -0.67 | 1.5 |
| WDR1 | -5.21 | -4.54 | -0.69 |
| cDNA FLJ61482 | -4.57 | -4.61 | 0.01 |
| RPL14 | -4.38 | -4.01 | -0.37 |
| GSTM1 (fragment) | -2.79 | -2.88 | 0.1 |
| LGALS7 | -2.54 | -1.63 | -0.91 |
| EGLN1 | -2.28 | -1.4 | -0.88 |
| MT1G | -2.03 | -1.49 | -0.54 |
| HLA-DRB1 | -1.99 | -1.27 | -0.72 |
| OPA1 | -1.7 | -1.51 | -0.19 |
| MTAP | -1.58 | -1.87 | 0.29 |
| HLA-A (fragment) | -1.54 | -1.87 | 0.33 |
| CALB1 | -1.48 | -1.62 | 0.14 |
| SPG11 | -1.01 | -1.1 | 0.09 |
| MIC60 | -1.01 | -1.35 | 0.34 |
| HLA-A | -1 | -1.11 | 0.12 |
| RAB6A | 1.1 | 1.45 | -0.34 |
| HEL113 | 1.36 | 1.1 | 0.25 |
| GSTM4 | -2.99 | -0.75 | -2.24 |
| MT1H | -2.39 | -0.6 | -1.8 |
| HLA-A (fragment) | -2.26 | -0.92 | -1.34 |
| HLA-B (fragment) | -2.11 | 0.68 | -2.8 |
| HLA-B | -2.06 | 0.59 | -2.65 |
| HLA-DPA1 | -1.91 | 0.43 | -2.34 |
| PRG3 | -1.87 | -0.25 | -1.62 |
| PIGQ | -1.86 | -0.2 | -1.67 |
| PRTN3 | -1.81 | 0.16 | -1.97 |
| Uncharacterized protein (Fragment) | -1.74 | 0.88 | -2.62 |
| HLA-DRB1 (fragment) | -1.69 | 0.04 | -1.72 |
| TRG14 | -1.64 | 0.45 | -2.1 |
| IFITM1 | -1.58 | -0.25 | -1.33 |
| PRG2 | -1.46 | 0.45 | -1.91 |
| ALDH7A1 | -1.46 | -0.42 | -1.04 |
| GATM | -1.45 | 0.1 | -1.55 |
| KIAA1671 | -1.43 | -0.09 | -1.34 |
| VPS11 | -1.41 | 0.41 | -1.83 |
| ALDOC | -1.41 | 0.39 | -1.8 |
| EPX | -1.39 | 0.18 | -1.57 |
| FGB | -1.35 | -0.19 | -1.16 |
| CST7 | -1.33 | 0.31 | -1.65 |
| cDNA FLJ93141 | -1.33 | 0.15 | -1.48 |
| HLA-C | -1.3 | 0.32 | -1.62 |
| FLJ00293 | -1.3 | 0.12 | -1.42 |
| PPBP | -1.27 | 0.22 | -1.49 |
| FAM89A | -1.27 | 0.1 | -1.37 |
| MFGE8 | -1.25 | 0.07 | -1.31 |
| FGG | -1.25 | -0.19 | -1.06 |
| ITGB3 | -1.23 | -0.01 | -1.23 |
| MGST1 | -1.23 | -0.06 | -1.17 |
| HLA-A (fragment) | -1.19 | 0.41 | -1.6 |
| QPRT | -1.19 | 0.21 | -1.39 |
| HVCN1 | -1.15 | 0.72 | -1.87 |
| AK4 | -1.14 | 0.08 | -1.21 |
| TSPAN25 | -1.11 | 0.31 | -1.42 |
| HMGCS1 | -1.1 | 0.4 | -1.49 |
| cDNA FLJ61355 | -1.09 | 0.02 | -1.11 |
| PRSS57 | -1.07 | 0.89 | -1.96 |
| P4HA1 | -1.07 | 0.55 | -1.62 |
| SLC16A3 | -1.06 | 0.58 | -1.64 |
| BNIP3 | -1.06 | 0.41 | -1.47 |
| NCF4 | -1.03 | 0.1 | -1.13 |
| cDNA FLJ93263 | -1.03 | 0.06 | -1.09 |
| PFKFB4 | -1.01 | 0.3 | -1.32 |
| BASP1 | -1 | 0.33 | -1.33 |
| TXNIP | -1 | 0.31 | -1.31 |
| C3 | 1.01 | 0 | 1.01 |
| PDCD11 | 1.01 | -0.06 | 1.06 |
| cDNA FLJ76826 | 1.03 | -0.14 | 1.18 |
| GCT-A4 light chain variable region (Fragment) | 1.03 | -0.22 | 1.24 |
| NOL6 | 1.03 | -0.31 | 1.34 |
| HBZ | 1.04 | -0.03 | 1.07 |
| MS-D4 heavy chain variable region (Fragment) | 1.06 | -0.16 | 1.22 |
| cDNA FLJ78497 | 1.1 | 0.01 | 1.09 |
| Lambda-chain (AA -20 to 215) | 1.1 | -0.13 | 1.23 |
| CDK105 | 1.11 | 0.09 | 1.02 |
| cDNA FLJ55606 | 1.12 | 0.11 | 1.01 |
| VTN | 1.12 | -0.08 | 1.2 |
| INS | 1.12 | -0.46 | 1.01 |
| WDR87 | 1.14 | -0.16 | 1.3 |
| Uncharacterized protein (A8K008_HUMAN) | 1.14 | -0.28 | 1.42 |
| HP | 1.15 | -0.13 | 1.28 |
| IGL@ | 1.16 | -0.21 | 1.36 |
| cDNA FLJ93914 | 1.18 | -0.4 | 1.57 |
| AKR1C3 | 1.19 | -0.2 | 1.4 |
| cDNA FLJ61695 | 1.19 | -0.29 | 1.48 |
| ORM1 | 1.22 | -0.05 | 1.28 |
| KNG1 | 1.25 | 0.11 | 1.14 |
| FAU | 1.27 | -0.68 | 1.95 |
| DKFZp686J11235 (Fragment) | 1.28 | -0.12 | 1.4 |
| GC | 1.29 | -0.05 | 1.34 |
| AATF | 1.33 | 0 | 1.33 |
| SERPINA1 | 1.33 | -0.09 | 1.42 |
| NUDT22 | 1.36 | -0.21 | 1.57 |
| CAND2 | 1.37 | 0.29 | 1.09 |
| SERPINA1 | 1.38 | -0.06 | 1.44 |
| TTR | 1.4 | 0.03 | 1.37 |
| PRDX3 | 1.4 | -0.32 | 1.72 |
| GNL3 | 1.47 | -0.02 | 1.48 |
| ORM2 | 1.5 | 0.04 | 1.46 |
| UTP11 | 1.51 | 0.2 | 1.31 |
| HLA-A (fragment) | 1.55 | 0.26 | 1.29 |
| cDNA FLJ41981 fis | 1.58 | 0.01 | 1.57 |
| HBE1 | 1.74 | 0.24 | 1.5 |
| ZNF614 | 1.81 | 0.05 | 1.76 |
| HPR | 1.92 | 0.49 | 1.43 |
| APOC3 | 2.09 | 0.19 | 1.9 |
| APOA1 | 2.44 | 0.94 | 1.49 |
| HLA-A (fragment) | 3.45 | 0.6 | 2.85 |
| Mutant HBB (fragment) | 0.22 | -5.8 | 6.04 |
| MYO10 | -0.22 | -2.35 | 2.13 |
| LBR | -0.04 | -2.3 | 2.26 |
| LIMS4 | -0.28 | -1.98 | 1.7 |
| SERPINB5 | -0.4 | -1.69 | 1.28 |
| cDNA FLJ57476 | 0.1 | -1.51 | 1.62 |
| DCD | -0.31 | -1.39 | 1.08 |
| BRMS1L | -0.19 | -1.19 | 1 |
| GAPDH (fragment) | -0.61 | 1.03 | -1.63 |
| cDNA FLJ54845 | -0.47 | 1.11 | -1.58 |
| PTPNS1 | -0.67 | 1.18 | -1.85 |
| cDNA FLJ77874 | -0.21 | 1.18 | -1.38 |
| CYP20A1 | 0.12 | 1.18 | -1.06 |
| OXA1L | -0.15 | 1.2 | -1.35 |
| SRRM1 | -0.51 | 1.54 | -2.04 |
| cDNA FLJ59273 | 0.12 | 1.77 | -1.66 |
| GSTT1 | -0.51 | 1.78 | -2.29 |
| DKFZp686M0430 | 0.85 | 2.48 | -1.63 |
| cDNA FLJ75556 | 0.3 | 2.49 | -2.19 |
| GMNN (variant fragment) | -0.16 | 3.12 | -3.28 |
| ACADS | -0.99 | 3.23 | -4.21 |

**Table S2** Numbers of proteins differentially expressed in pairwise comparison

| Comparison | Numbers of protein (sample that they were expressed more abundantly in) | | Total numbers of differentially expressed protein |
| --- | --- | --- | --- |
| PVN vs. CT | 71 (PVN) | 78 (CT) | 149 |
| PVN vs. PVT | 27 (PVN) | 36 (PVT) | 63 |
| PVT vs. CT | 86 (PVT) | 137 (CT) | 223 |

**
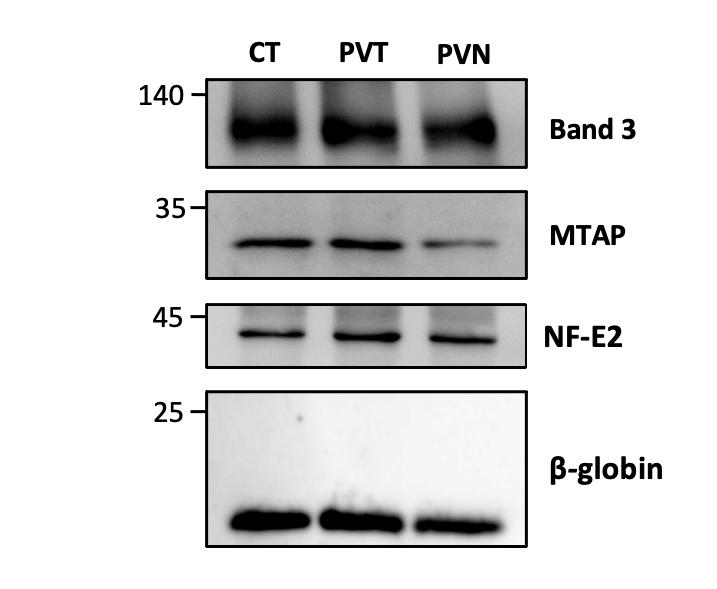
**

**Figure S1: Levels of MTAP, NF-E2, Band 3, and β-globin in PVT and PVN patient erythroid cells compared to control.** Western blot was performed on the pooled samples of day 7 erythroblasts from CT, PVT, and PVN groups (n=3).


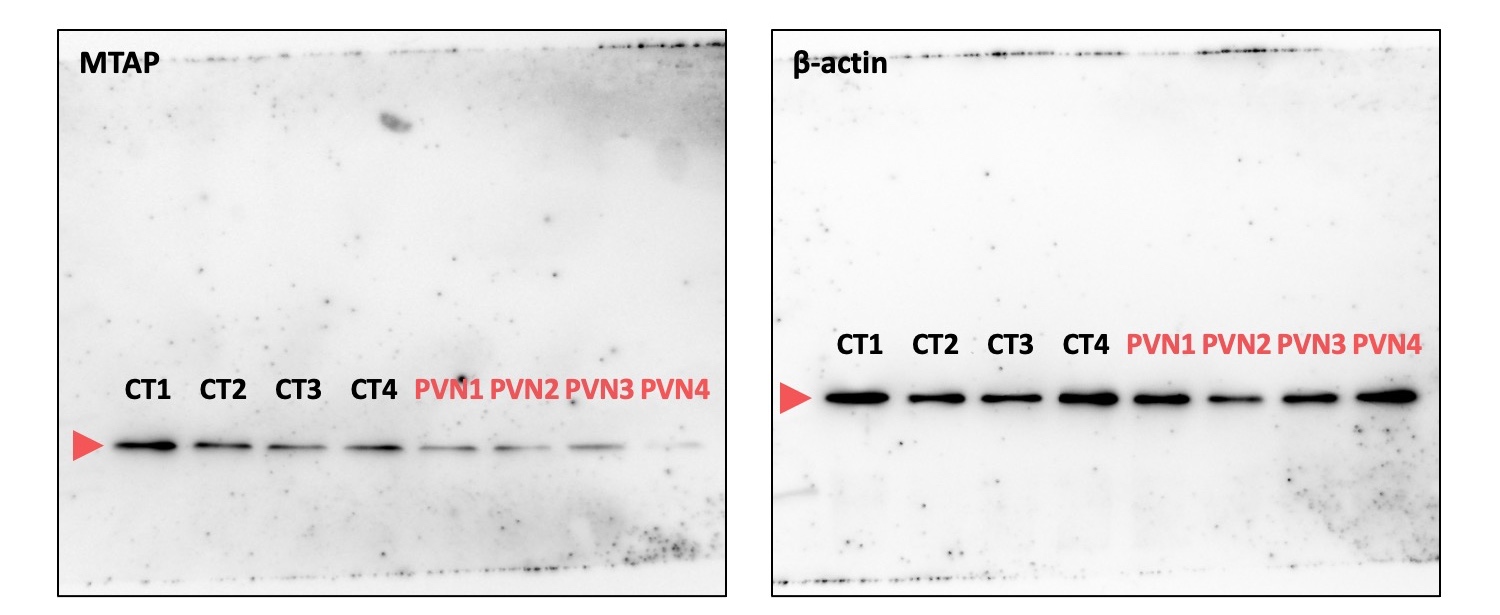


**Figure S2: Expression of MTAP and β-actin (control) determined by western blot.** Full-length blots of images shown in Figure 4A.

**
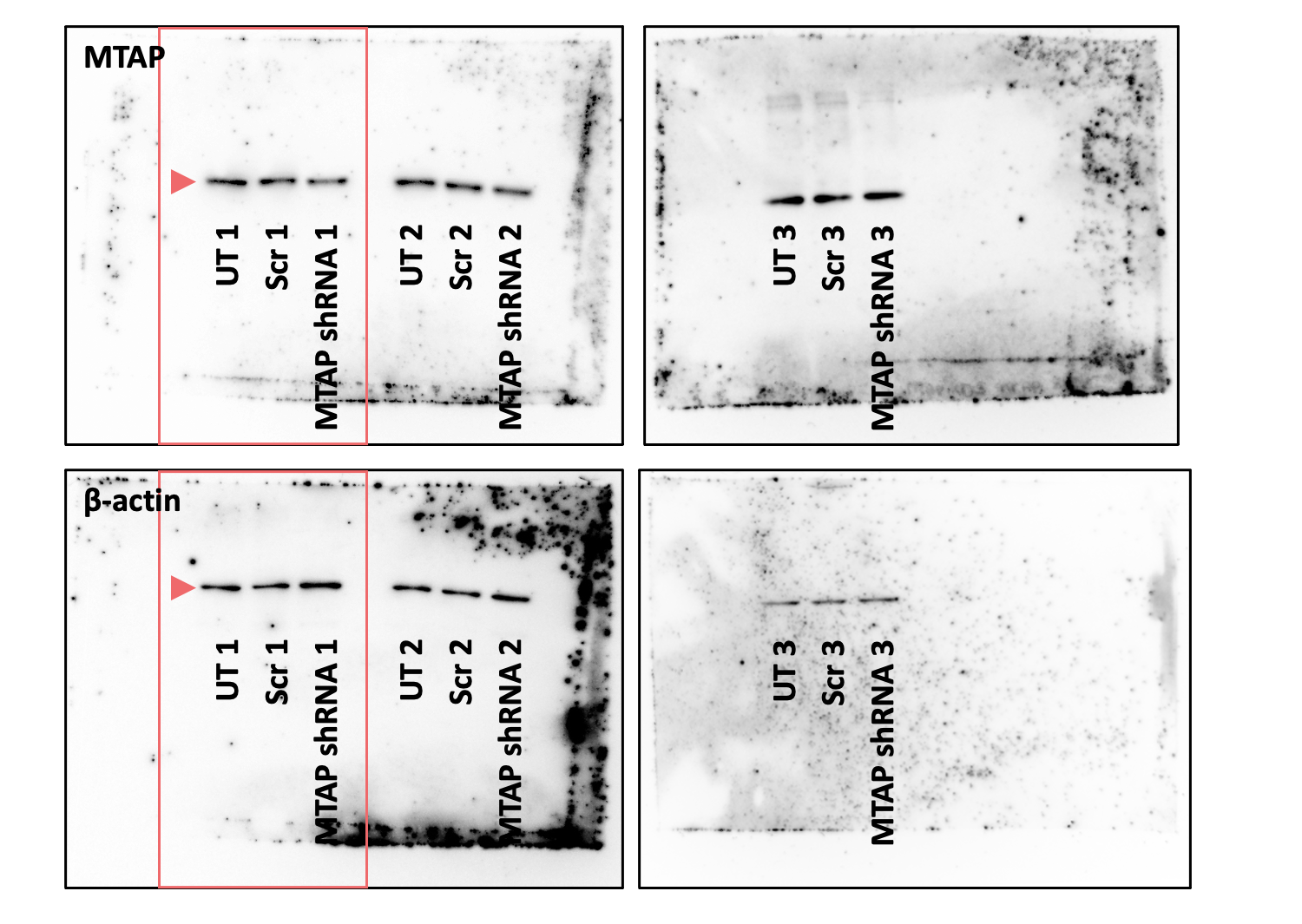
**

**Figure S3: Expression of MTAP and β-actin (control) determined by western blot.** Full-length blots of images shown in Figure 4C (red rectangles indicated lanes shown in Figure 4C). Other lanes in the blots were replicates that were included in the calculation for Figure 4D.

**
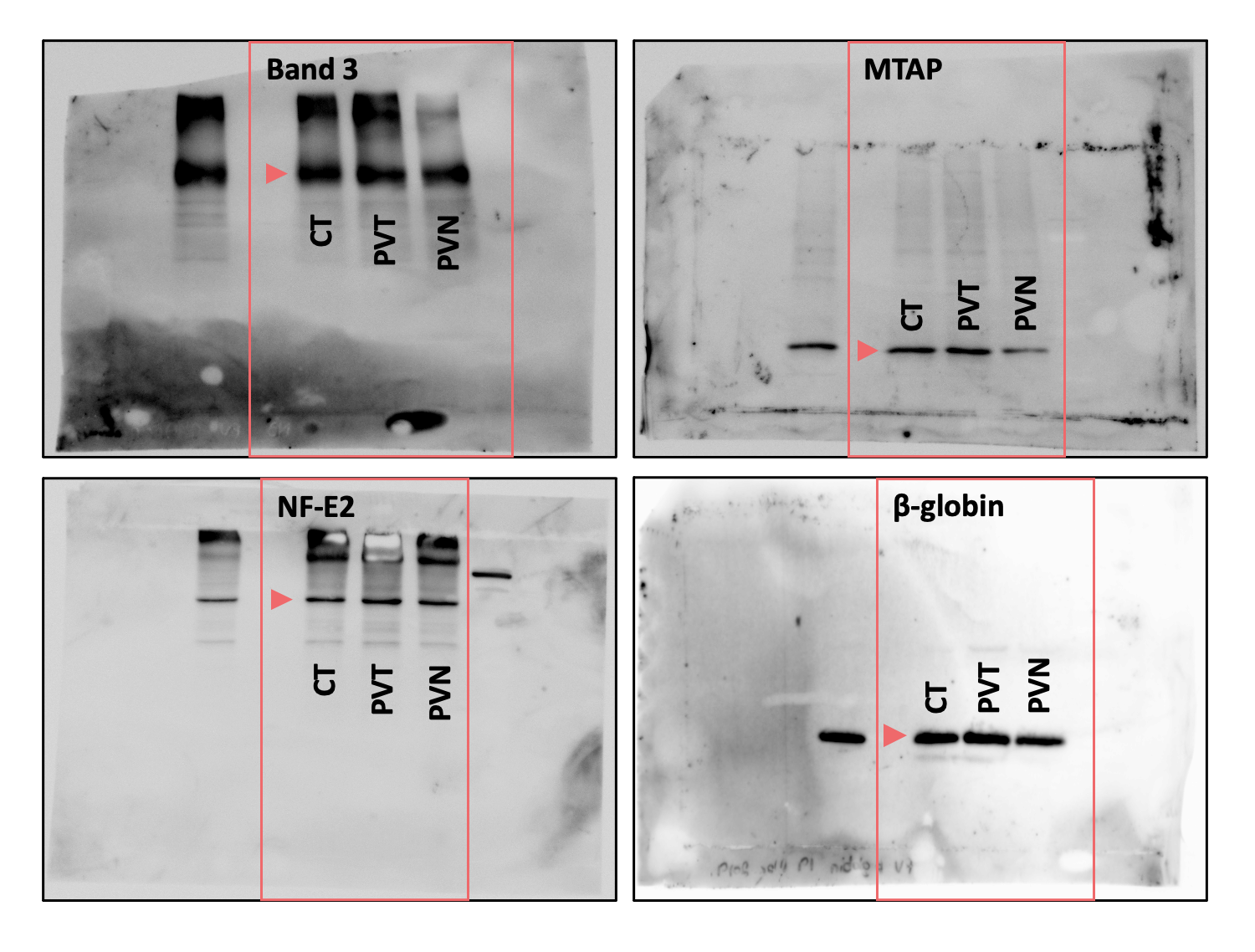
**

**Figure S4: Expression of MTAP, NF-E2, Band 3, and β-globin in PVT and PVN patient erythroid cells compared to control determined by western blot.** Full-length blots of images shown in Figure S1 (red rectangles indicated lanes shown in Figure S1).
